# Enkephalins: Endogenous Analgesics with an Emerging Role in Stress Resilience

**DOI:** 10.1155/2017/1546125

**Published:** 2017-07-11

**Authors:** Mathilde S. Henry, Louis Gendron, Marie-Eve Tremblay, Guy Drolet

**Affiliations:** ^1^Axe Neurosciences, Centre de Recherche du CHU de Québec – Université Laval, Québec, QC, Canada; ^2^Département de pharmacologie-physiologie, Centre de Recherche du CHU de Sherbrooke, Institut de Pharmacologie de Sherbrooke, Université de Sherbrooke, Sherbrooke, QC, Canada; ^3^Quebec Pain Research Network, Sherbrooke, QC, Canada

## Abstract

Psychological stress is a state of mental or emotional strain or tension that results from adverse or demanding circumstances. Chronic stress is well known to induce anxiety disorders and major depression; it is also considered a risk factor for Alzheimer's disease. Stress resilience is a positive outcome that is associated with preserved cognition and healthy aging. Resilience presents psychological and biological characteristics intrinsic to an individual conferring protection against the development of psychopathologies in the face of adversity. How can we promote or improve resilience to chronic stress? Numerous studies have proposed mechanisms that could trigger this desirable process. The roles of enkephalin transmission in the control of pain, physiological functions, like respiration, and affective disorders have been studied for more than 30 years. However, their role in the resilience to chronic stress has received much less attention. This review presents the evidence for an emerging involvement of enkephalin signaling through its two associated opioid receptors, *μ* opioid peptide receptor and *δ* opioid peptide receptor, in the natural adaptation to stressful lifestyles.

## 1. Introduction

Psychological stress is a state of mental or emotional strain or tension that results from adverse or demanding circumstances. It has multifaceted causes and occurs frequently over a lifetime with varying dimensions and intensity, affecting all walks of life, irrespective of a person's occupation or position within a society [1]. While depression is often the devastating outcome of chronic stress [2] and also a risk factor and common comorbidity in Alzheimer's disease [3, 4], stress resilience, on the other hand, is a positive outcome that is associated with preserved cognition, reduced oxidative damage, and healthy aging [5, 6]. The American Psychological Association defines resilience as “the process of adapting well in the face of adversity, trauma, tragedy, threats or even significant sources of threat.” Heterogeneity in the response to chronic stress suggests that resilience is a complex neurobiological process that emerges from a multitude of gene-environment interactions. Several mechanisms are proposed to underlie the interindividual differences in resilience or vulnerability to chronic stress.

Within the neuropeptidergic system, the endogenous opioids enkephalins (ENK) which signal through the opioid peptide receptors (OPr), *μ* opioid peptide receptor (MOPr) and *δ* opioid peptide receptor (DOPr), could be interesting candidates to naturally promote the adaptation to chronic stress. ENK are members of the endorphin family and the first ones to be isolated in the brain [7]. Considering the binding of morphine and ENK to the same receptors, their role as a natural analgesic was rapidly proposed. Pioneered studies have provided the first experimental evidence supporting a role of ENK in analgesia and stress-induced analgesia (i.e., pain suppression after an exposure to stressful stimuli). More specifically, it was shown in the rat that 1) the cerebroventricular injection of ENK produces analgesia [8, 9]; 2) stress increases blood concentrations of ENK [10]; and 3) stress-induced analgesia, such as immobilization stress on a hot plate or cold water stress, could be reversed by an opioid antagonist [11, 12]. Subsequently, it was hypothesized that ENK were playing a major role in stress processes independently of their analgesic functions. Madden et al. reported that inescapable stress induced by footshocks (mimicking a posttraumatic stress disorder; PTSD) increases brain levels of ENK [10]. Another study showed a decrease of ENK immunoreactivity in the rat hypothalamus (HPT) after stress induced by footshocks [13]. More recently, ENK in the rat amygdala (AMG) were implicated in Pavlovian conditioned fear [14, 15] as well as in various behavioral and neuroendocrine aspects of the stress response [16–18]. The ENK are known to be involved in a large set of physiological and emotional processes, but their role in the individual capacity for stress adaptation has received less interest. In this review, the biochemistry of ENK and their anatomical distribution within the central nervous system (CNS) will be described first, followed by coverage of the well-known functions of ENK in emotional behaviors, including their key involvement in Pavlovian conditioned fear, anxiety, and stress response. Subsequently, the emerging role of ENK in the development of stress resilience will be discussed, with an emphasis on the recruitment of ENK projections coming from the AMG. The AMG is considered a key brain structure mediating the regulation of emotions and affective behavior, and the role of ENK in the stress response is notably suggested by their extended distribution in the AMG.

## 2. Enkephalins and Their Opioid Receptors

### 2.1. Biochemistry and Anatomical Distribution of Enkephalins and Their Receptors, DOPr and MOPr, in the CNS

ENK are produced from a propeptide precursor, proenkephalin (proENK), which is translated from preproenkephalin mRNA that is encoded by a gene distinct from the other endogenous opioid peptides [19, 20]. The maturation of propeptides into functional peptides is performed during the vesicular transport within large dense-core vesicles (LDCVs) and requires the joint action of several endopeptidases (cathepsin L, aminopeptidase B and E, and prohormone convertase 2) [21–23]. In the rat, the proENK is cleaved proteolitycally to produce four copies of methionine-ENK (Met-ENK), one leucine-ENK (Leu-ENK), and two C-terminal extended Met-ENK. Subsequently, LDCVs are stored near release sites (i.e., presynaptic, extrasynaptic, and dendritic) and released following an increase in intracellular calcium [24]. Once released by neurons, ENK are degraded in order to control the diffusion and synchrony of the signal. Some studies demonstrated that radioactively labeled ENK are completely degraded in less than a minute upon injection (intracerebroventricular) in the rat brain [25]. ENK degradation is performed by two neuropeptidases called metallopeptidases: aminopeptidase N and neutral endopeptidase (or neprilysin) [26, 27]. In vitro, ENK have a slightly higher affinity for DOPr, even though they can also bind and activate MOPr and *κ* opioid peptide receptor (KOPr) in transfected cells transiently expressing MOPr, DOPr, or KOPr [28]. Studies describing the distribution of ENK in the rat brain have demonstrated their preferential binding to DOPr and MOPr by autoradiographic labelling [29].

Given the vast extent of biological processes and physiological systems in which ENK are involved (cardiovascular system, thirst and feeding, pain and analgesia, gastrointestinal functions, respiration, etc. [30]), the expression of ENK, DOPr, and MOPr is ubiquitous. Indeed, ENK are distributed among the central, peripheral, and autonomous nervous systems, as well as in endocrine tissues (adrenal medulla, endocrine pancreas) and their target organs (liver, skin, bones, and lungs) [31, 32]. For the purpose of this review, we will focus mainly on the neuroanatomical distribution of ENK and their receptors within the “emotional brain” known as the limbic system that includes the cingulate and entorhinal cortex, hippocampus (HPC), septum, HPT, and the extended AMG [33]. Most of neuroanatomic studies have been conducted in rats, although several studies have also been conducted in humans, showing a similar distribution across species, especially in the limbic system [34]. Fallon and Leslie extensively reported in 1986 the distribution of ENK neurons as well as ENK fibers in the rat brain using an indirect immunofluorescence technique [35]. ENK neurons are found among the entorhinal, piriform, and medial prefrontal cortex (mPFC, infralimbic and prelimbic). Most nuclei of the HPT were shown to contain ENK neurons (paraventricular, posterior, ventromedial, dorsal, dorsomedial, and lateral nuclei). They are widely distributed in the central (CEA), medial (MEA), and basolateral (BLA) AMG and its intercalated (IC) nuclei. ENK neurons are also located in the lateral septum, preoptic area, bed nuclei of the stria terminalis (BST), nucleus accumbens (NAc), and ventral tegmental area (VTA). In the HPC, ENK are present in mossy fibers and granular cells. ENK fibers mainly project from the dentate gyrus to the CA3 region of Ammon's horn, but also target some neurons of the CA1 and CA2, and dentate gyrus. Additionally, ENK fibers are found in the dorsal and ventral pallidum [35, 36].

Similar to ENK, OPr are extensively expressed throughout the CNS [37]. The anatomical distribution of MOPr and DOPr is relatively similar to that of ENK projections [38]. To study the relative distributions of MOPr and DOPr throughout the CNS, Scherrer and colleagues have generated a very useful mouse model. They first developed DOPr-eGFP *knock in* (KI) mice, presenting a complete functional receptor fused to an enhanced green fluorescent protein (eGFP) [39]. These mutant mice were subsequently crossed to another model containing a similar construct, MOPr-mcherry KI mice [40]. This breeding generated a double KI mouse useful for in situ visualization of DOPr and MOPr simultaneously [40, 41]. The study of DOPr and MOPr distribution in the CNS showed that coexpression of DOPr and MOPr is observed in HPT, HPC, the lateral parabrachial nucleus and vestibular nuclei, circuitries which are involved in survival including water and food consumption, sexual behavior, and response to aversive stimuli [40]. The large distribution of ENK and their associated receptors in the limbic system of rodents and humans further suggests that ENK transmission plays a major role in emotional behaviors.

### 2.2. Roles in Emotional Behaviors

ENK are indeed involved in several emotional behaviors, including fear conditioning [14, 15, 42–45], anxiety, and stress response [46–65]. This section will describe the experimental evidence for such a role, mainly derived from studies conducted in rodents, using different approaches, neuroanatomical, silencing, pharmacological, and genetic, as well as stress paradigms varying in chronicity and intensity.

#### 2.2.1. Fear Conditioning

The fear conditioning paradigm allows assessment of learning and memory in association with fear (see Table 1). The first evidence that ENK participate in fear conditioning comes from an in situ hybridization study showing an increase in ENK mRNA levels in the CEA neurons of rats undergoing this paradigm [14]. Thereafter, it was shown that ENK *knockout* (KO) mice exhibit an exaggerated immobility compared to wild-type controls during the auditory-conditioned fear acquisition [42]. A population of GABAergic neurons expressing protein kinase C-*δ* (PKC-*δ*) was identified in the lateral part of CEA (CEAl), using a molecular genetic approach in mice. Interestingly, this population appears to overlap with ENK neurons [45]. In another study, it was shown that this neuronal population expressing PKC-*δ* in the CEAl is implicated in the inhibition of fear acquisition [66]. However, the exact role of ENK expressed by these PKC-*δ* GABAergic neurons is still undetermined.

Asok et al. also showed that exposure to a component of fox odor, 2,5-dihydro-2,4,5-trimethylthiazoline (TMT), which triggers innate fear in rats, increases ENK mRNA levels in the paraventricular nucleus (PVN) of the HPT [43]. An increased expression of ENK mRNA levels is similarly observed after repeated footshocks, in the AMG of SWR/J mice, an inbred strain showing a reduced fear response, while this expression was unchanged in C57Bl/6J mice, an inbred strain showing a high fear response [44]. In the same study, administration of MOPr antagonist (naltrexone) or DOPr antagonist (naltrindole) increased fear response in SWR/J mice, which could be restored with a DOPr agonist. These results suggest that resistance in the face of traumatic experiences inducing fear involves ENK from the AMG and that vulnerability can be modulated by administration of OPr agonists [44]. Finally, it has been shown by Poulin et al. that the downregulation of ENK in the rat CEA decreases unconditioned fear [15]. In this study, rats were submitted to a contextual conditioning paradigm consisting of footshocks administered in a novel environment. ENK *knockdown* (KD) rats showed a reduced fear response during conditioning, while the context alone, presented 48 h later, did not produce change in freezing behavior. These results indicate that ENK release from CEA neurons is involved in the freezing behavior to an unconditioned stimulus, but not in the formation of an associative memory [15]. Results of ENK distribution studies—in addition to pharmacological, silencing, and genetic studies—demonstrate the prominent role of ENK, especially amygdalar ENK, in mediating fear behavior. This connection may further suggest a role for ENK in anxiety and stress responses, which are closely related to fear behavior.

#### 2.2.2. Stress and Anxiety

Several studies performed in humans showed the importance of ENK in anxiety, depression, and PTSD, a mental illness that appears after experiencing a traumatic event. Indeed, a polymorphism in the gene encoding neutral endopeptidase, involved in ENK metabolism, was identified in patients with anxiety disorder, tested with the SCL-90-R inventory of psychological symptoms [48]. Positron emission tomography (PET) studies have shown that MOPr expression is decreased in the anterior cingulate cortex of patients with PTSD [47]. In patients with depression, PET further revealed that the expression of MOPr is decreased in the HPT and AMG [49]. These studies suggest that a reduced tone of ENK neurotransmission is a key component in the expression of anxiety.

In rodents, several behavioral paradigms are commonly used to assess the level of anxiety, including the elevated plus maze (EPM), open field (OF), and light-dark box (LDB) tests. These tests are based on the natural aversion of rodents for open, elevated, or illuminated areas and their natural exploratory behavior in novel environments. In addition, the social interaction test (SI) allows evaluating the propensity to socialize. The startle response (SR) corresponds to an unconscious defensive response to unexpected or threatening stimuli. All behavioral tests discussed in our review are detailed in Table 1.

The ENK KO mice show an increased anxiety with the EPM, OF, and LDB tests, have an exaggerated SR, and a reduced duration of SI [42, 50, 51]. ENK KO mice exposed to a stress induced by footshocks, mimicking PTSD, similarly present anxiety- and depressive-like behaviors, contrary to wild-type controls, using the OF, EPM, and LDB tests (see Table 1) [52]. However, the downregulation of ENK in CEA was shown to reduce anxiety as characterized by an increase of exploratory behavior [15]. ENK KO mice are resistant to anxiety- and depression-like behaviors after a chronic mild unpredictable stress—consisting of daily exposure to different stressors, such as food deprivation and restraint stress for five weeks—suggesting that ENK enhance the reactivity to chronic stress [67]. ENK appear to have varying and even opposing effects on anxiety, depending on the considered CNS region and the type and intensity of stress.

The high levels of anxiety generally observed in ENK KO mice are also seen upon gene inactivation of DOPr [53]. Pharmacological studies conducted in rodents support these results obtained through gene inactivation of DOPr, since subcutaneous administration of naltrindole, a DOPr antagonist, induces anxiety [54]. Conversely, intraperitoneal injection of DOPr agonists (SNC80, UFP-512, (+)BW373U86) was shown to be anxiolytic [55–57]. Moreover, infusion of [D-Pen 2,5]-ENK (DPDPE), a DOPr agonist, in CEA exerted similar effects, which could be reversed by the administration of naltrindole, a DOPr antagonist. Recently, a new DOPr agonist, KNT-127, has received an increasing interest as a potential therapeutic treatment for anxiety and depression, although the efficacy of this molecule has not yet been investigated in clinical trials. In rodents, KNT-127 produces anxiolytic and antidepressant-like effects in a dose-dependent manner (see Table 1) [59, 60]. These results are consistent between models and suggest that signaling onto DOPr mainly exerts anxiolytic effects.

In contrast to these findings, a conditional KO mouse for DOPr (Dlx-DOR) in forebrain GABAergic neurons showed a reduced level of anxiety compared to wild-type littermates, demonstrating that stimulation of DOPr in GABAergic neurons of the forebrain is anxiogenic (see Table 1) [62]. In the same way, the gene inactivation of MOPr has anxiolytic effects, with MOPr KO mice presenting an increased time spent in the open arms of an EPM [53]. Nevertheless, several pharmacological studies instead demonstrated that MOPr activation is anxiolytic. For example, intraperitoneal administration of morphine, a MOPr agonist, decreases vocalizations in rats exposed to a predator and anxiety assessed with the EPM test [58]. Overall, MOPr appear to have varying effects on anxiety, depending on the methodological approaches used.

A few recent studies explored the neuroanatomical specificity of ENK projections that are recruited in steady-state conditions or upon stress in rats. Single housing (see Table 2) in early life was shown to decrease immunoreactivity of Met-ENK-Arg^6^Phe^7^ (MEAP) in the brain areas that include the AMG, substantia nigra (SN), HPT, and periaqueductal grey (PAG) [63]. Hernández et al. also measured ENK neuropeptidase activities in the three main regions of the stress response circuitry (AMG, HPC, and mPFC) after acute restraint in rats (see Table 2; [46]). Neuropeptidases regulate the expression of neuropeptides at the release sites. Peptidase activity can thus be used to indicate the functional status of neuropeptides. This neuropeptidase activity was found to be more intense in AMG than in HPC or mPFC both in control and stressful conditions, suggesting that ENK metabolism is preponderant in the AMG. After acute restraint stress, ENK-degrading activity was reduced in AMG and increased in HPC, while it remained unchanged in the mPFC. In stressed rats, a positive correlation was described between the AMG and HPC, while in control rats, a negative correlation was observed between the mPFC and HPC. These results suggest a neuropeptidergic functional connection between the mPFC, HPC, and AMG, which could be triggered by stress and involved in some of the adaptive functions performed by this circuit.

Overall, these contradictory results found in the literature regarding the influence of ENK signaling on anxiety could be attributed first, to the technical approaches (pharmacological, genetic), then to the considered nucleus (CEA for example) or associated neurotransmitters (GABA), and finally to the type (acute, chronic stress) and intensity of stress. It still remains unknown whether the many effects of ENK circuitry acting in such a diverse array of brain circuits might all be recruited together in response to a variety of different stressors and different modalities. Different brain circuits could synergistically contribute to the stress response, highlighting the huge challenge we face in understanding the functions of ENK signaling. Taken together, the combined findings from these silencing, pharmacological, genetic, and neuroanatomical studies suggest that the stimulation of ENK transmission onto DOPr and/or MOPr might enhance the natural strategies to cope with stress.

## 3. Enkephalin Signaling through DOPr and MOPr, a Major Component of the Stress Resilience Circuitry

An extreme amount of stress can lead to maladaptive behavioral changes such as anhedonia and social avoidance, in rodents and humans, as well as serious health consequences by impacting on the nervous, endocrine, and immune systems. However, chronic exposure to stress can also engender compensatory physiological responses in order to reduce these deleterious effects of stress. This mechanism of defense allows maintaining homeostasis in the face of adversity. This phenomenon of “resilience” corresponds to the ability of an individual to maintain normal psychological and physical functioning in the front of stress or trauma, in order to avoid mental and physical illnesses [68].

Recent findings regarding the functions of ENK transmission in stress resilience revealed the involvement of different brain areas such as the NAc [69, 70] or septum, PVN and PAG [17, 18, 71], or locus coeruleus (LC) and paragigantocellularis nucleus (PGi) [72], in addition to the BLA as we will discuss below, thus suggesting a high level complexity of ENK circuitry in stress resilience.

Sweis et al. associated the resilience to chronic stress—measured by a lack of memory impairment poststress—to an increased expression of ENK mRNA in the rat NAc, proposing that an ENK-mediated increase of dopaminergic tone could improve motivation-based cognitive performance [69]. This predominant role of ENK projections from the NAc is supported by the results of another study. Indeed, it was shown that after 14 days of restraint stress, rats showing increased anhedonia (as measured by their preference for sucrose—see Table 1) also presented in the NAc a reduced expression of ENK mRNA and ΔFosB, a transcription factor that is expressed by ENK neurons. These results suggest that the individual vulnerability to chronic stress, determined here by measuring anhedonia, is associated with a ΔFosB-mediated downregulation of ENK [70]. The relationship between ΔFosB and the resilience to chronic stress was already known [73].

Downstream of ENK, Akil et al. also studied in rats the effects of dominance status and housing conditions on the response to a DOPr agonist, SNC80 [74]. This study revealed that single housing for 50 days leads to a stronger DOPr activation in the mPFC, CEA, and NAc. Triad housing for the same period of time also increases DOPr activation in the mPFC, CEA, and NAc, in addition to the median eminence and thalamus, of *β* rats that we can assimilate to stress resilient individuals considering their defensive behaviors and frequent aggressive interactions with *α* dominant rats, which instead display an offensive behavior [74]. This mechanism could be involved in the regulation of ENK transmission upon stress.

Two types of behavioral paradigms are commonly conducted in rodents for studying stress resilience (see Table 2). The social defeat paradigm, also named resident-intruder paradigm, in which intruder animals are repeatedly submitted to daily interactions with a home-cage unfamiliar resident over a given period of time, induces stress resilience by mimicking the unpredictable social disruptions of daily life. This paradigm has been shown to present excellent etiological, predictive, discriminative, and face validity [75]. Moreover, unlike other stress paradigm, social defeat stress leads to long-lasting changes in hypothalamic-pituitary-adrenal axis function, making it a stress paradigm of choice [76]. The majority of rodents exposed to this paradigm exhibits reduced motivation, anhedonia, and avoids social interactions [77]. Conversely, despite the deleterious effects of social stress, around 30% of the population presents a phenotype of stress resilience, being resistant to the emergence of depressive-like behavior. In rats, the daily interaction between individuals results in subordination of the intruder, indicated by adoption of a supine position. The latency to assume a defeated posture is recorded, and the averaged latency over stress exposition is used as a predictive value to define resilience or vulnerability to stress. In mice, the resilience or vulnerability to stress is instead assessed at the end of this experiment by using a SI test (see Table 1). The second chronic stress paradigm that is commonly used to study stress resilience is chronic unpredictable stress. In this experiment, individuals are daily submitted to different stressors that include restraint stress, wet bedding, food deprivation, and footshocks. The phenotype of resilience or vulnerability to stress is assessed at the end of the experiment in mice and rats using behavioral tests previously described such as SI, EPM, and OF (also see Table 1).

For example, Briand et al. used the repeated social defeat paradigm in a mouse model of OPRM1 A118G polymorphism (single nucleotide polymorphism, SNP) corresponding to a genetic mutation of MOPr observed in humans that is associated with an overall reduction of baseline MOPr availability in regions implicated in pain and affective regulation [78], thus allowing to unravel a potential role of MOPr in the resilience to chronic stress [71]. This model presents increased home-cage dominance and nonaggressive social interactions, similar to the human carriers of this mutation. In the presence of an aggressor during social defeat stress, it also showed a strong resilience to chronic stress, determined by a blunted anhedonia and social avoidance following the social defeat. Neuronal activation measured by c-fos staining was additionally increased in the NAc, septum, BLA, PVN, and PAG, thus suggesting an increased release of endogenous opioids upon stress [71]. In humans, Troisi et al. demonstrated that the carriers of this mutation have a greater capacity to experience social reward and are more prone to fearful attachment, a personality trait that is related to rejection sensitivity, regardless of the quality of maternal care [79, 80].

Reyes et al. also revealed involvement of the ENK circuitry between the LC and PGi in stress resilience in rats. In this study, fluorogold, a retrograde tracer, was injected into the LC to determine involvement of different afferents (corticotropin-releasing factor, CRF neurons from CEA and ENK neurons from PGi) in resilience under the resident-intruder paradigm [72]. Individuals presenting a reduced latency to present a defeated posture (defined as vulnerable rats) showed an increased activation of LC neurons and afferents of CRF neurons from CEA. Conversely, resilient rats (longer latency to present a defeated posture) demonstrated a higher recruitment of ENK afferents from PGi. Thus, two different afferent pathways to the LC, from CRF neurons in the CEA and ENK neurons from the PGi, would partly define the interindividual variation with regard to the capacity to resist chronic stress.

Two studies conducted in our laboratory demonstrated that resilience to social defeat and chronic unpredictable stress share common variations of expression among the ENK systems within specific brain regions in rats [17, 18]. ENK mRNA (transcripts) were quantified in 23 nuclei of the mPFC, NAc, dorsal striatum, and AMG. Only one significant difference between control, resilient, and vulnerable individuals was found in the BLA of vulnerable individuals; ENK mRNA levels were decreased in vulnerable rats compared to control and resilient rats. In contrast, no difference was found in ENK expression in the BLA between controls and resilient animals [17]. In addition to revealing these associations, the functional role of ENK in the AMG was evaluated. The downregulation of ENK in the BLA was shown to increase anxiety both in the SI test and EPM thus reproducing certain behavioral responses encountered in individuals that are vulnerable to chronic stress [18]. Finally, the chronic social defeat stress was conducted in mice in order to assess ENK signature in the BLA. The expression of ENK mRNA was found to be decreased by 33% in vulnerable mice, only in the BLA. No difference was found between the control and resilient individuals [81]. These combined results suggest that specific neuroadaptations mediated by ENK neurotransmission in the BLA could represent a key mediator of stress resilience. Based on these results, we can hypothesize that the decrease in ENK transmission from the BLA is a maladaptive mechanism, which mediates the behavioral dichotomy observed between vulnerable and resilient animals experiencing chronic stress.

## 4. Conclusion

**Figure 1:**
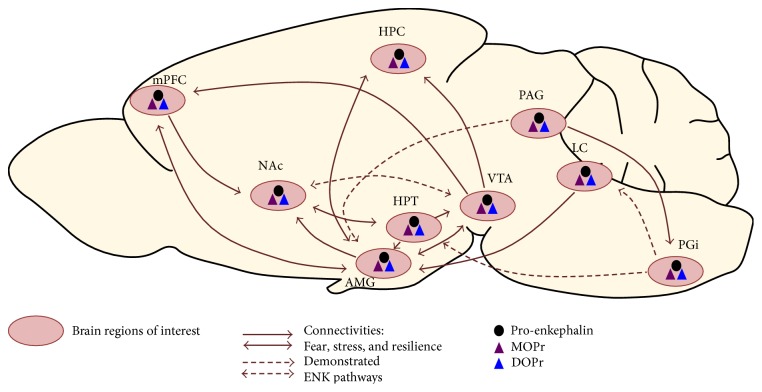
Cartography of main connectivities involved in fear, stress, and resilience, as well as demonstrated ENK pathways between areas and expression of ENK, MOPr, and DOPr. Pink circles represent brain regions of interest. Full arrows correspond to circuitries of stress, fear, and resilience. Dotted arrows represent demonstrated ENK circuitries. The black dot corresponds to expression of pro-enkephalin, and purple and blue triangles correspond to MOPr and DOPr expression, respectively. AMG: amygdala; HPC: hippocampus; HPT: hypothalamus; LC: locus ceruleus; mPFC: medial prefrontal cortex; NAc: nucleus accumbens; PAG: periaqueducal grey; PGi: paragigantocellularis nucleus; VTA: ventral tegmental area.

Overall, most of animal studies covered in this review suggest that ENK signaling could be targeted for promoting resilience to chronic stress. Resilience to chronic stress is a very complex process involving several brain structures and neurotransmitters. When considering only one neuropeptidergic system, the ENK acting through DOPr and MOPr, numerous implicated brain structures and circuits emerge (see Figure 1 for a schematic representation that we overlapped with the cartography of main connectivities known to be involved in stress response, fear, and resilience). While the roles of ENK signaling within certain brain structures such as AMG, HPT, and NAc were largely described, its involvement in other brain regions remains unknown with regard to stress resilience. For example, the preoptic area, BST, and piriform cortex express ENK without evidence for a potential role in resilience to chronic stress, to our knowledge. All of these circuits must be individually dissected. Complete ENK KO models may thus be inadequate for characterizing the involvement of ENK signaling in stress resilience. Hence, modulating ENK or DOPr/MOPr expression within circumscribed regions or modulating selected neuronal circuits appear to be more appropriate. In this regard, optogenetic tools could provide a unique opportunity to modulate ENK transmission among selected neuronal circuits, over the course of chronic stress and associated pathologies, as required to unravel the mechanisms through which distinct ENK pathways exert their functional role in stress resilience. Understanding the synergistic involvement of different circuits in stress resilience could additionally provide accurate, powerful, and effective therapeutic strategies to prevent or treat long-term anxiety and depression, in addition to a variety of stress- and anxiety-related disorders.

## Figures and Tables

**Table 1:** Evidence for ENK signaling involvement using different behavioral tests.

Behavior	Paradigm	Principles and procedures	Evidence for involvement of ENK signaling
Fear	Contextual fear conditioning	In this paradigm, an animal learns to predict aversive events based on their environmental context. It is a form of learning and memory in which an aversive stimulus is associated to a neutral context and/or stimulus, resulting in fear responses upon presentation of the originally neutral context and/or stimulus. The animal is placed into a chamber to administer an aversive stimulus (e.g., electric footshocks). This procedure can be paired with another conditioning stimulus, a sound for example. After a delay, the animal is reexposed to the environment and/or conditioning stimulus, without the aversive one. Freezing which is characterized by the total absence of movement except those required for respiration is then measured to assess fear responses.	(i) In rats, ENK mRNA levels are increased in CEA upon contextual fear conditioning [14] (ii) ENK *knockout* (KO) mice show an exaggerated immobility during auditory fear conditioning [42] (iii) ENK neurons in CEAl overlap with PKC-δ GABAergic neurons, which are involved in fear behavior [45, 66] (iv) In SWR/J mice (showing a reduced fear response induced by footshocks), ENK mRNA levels are increased in AMG [44] (v) In SWR/J mice (showing a reduced fear response induced by footshocks), administration of MOPr and DOPr antagonists increase fear response [44] (vi) In rats, ENK *knockdown* (KD) of CEA decreased unconditioned fear [15].
	Startle response	The startle reflex is considered as an innate and involuntary reaction that appears upon exposure to an unexpected or threatening stimuli. The response corresponds to a quick involuntary contraction of the animal's skeletal muscles. The test is conducted in an automated startle chamber that allows measurement of the reflex.	(i) ENK KO mice show an exaggerated startle response [50].

Anxiety	Open-field	This task is based on a rodent's preference for dark areas. The animal is placed in an open-field chamber, an arena with surrounding walls to prevent escape, and the exploratory behavior of the center (lit) versus periphery (dark) is assessed over time with a video-recording.	(i) ENK KO mice show a decreased exploratory behavior and avoid the central part of the open-field (OF) arena [42, 50, 51] (ii) ENK KO mice, exposed to stress induced by footshocks, present an anxiety-like behavior [52].
	Elevated plus maze	This task is based on a rodent's natural preference for dark and enclosed areas, compared to lit and uncovered areas, as well as on their natural exploratory behavior of a novel environment. The animal is placed in the maze, and its exploratory behavior is assessed over time with a video-recording. The maze has a cross shape with two opposite arms surrounded by walls (dark and enclosed area) whereas the two other arms do not present walls (lit and uncovered).	(i) ENK KO mice present anxiety-like behavior in the elevated plus maze (EPM) [50] (ii) ENK KO mice, exposed to stress induced by footshocks, present anxiety-like behavior in EPM [52] (iii) In rats, ENK KD in CEA increases the exploratory behavior in EPM [15] (iv) Infusion of a DOPr agonist in CEA increases the number of entries and the time spent in open arms of the EPM [82] (v) Administration of a DOPr antagonist diminishes the exploratory behavior in EPM [54] (vi) Administration of a DOPr agonist increases this behavior [55–57, 59, 60] (vii) DOPr KO mice spent less time in the open arms of EPM [53] (viii) MOPr KO mice increase the exploratory behavior in EPM [53] (ix) Administration of MOPr agonist increases the exploratory behavior in EPM [58].
	Light-dark box	This task is based on a rodent's natural preference for dark areas, compared to lit ones. The box contains two chambers, one light and one dark. The animal is placed into the box and its exploratory behavior is assessed over time with a video-recording.	(i) ENK KO mice show a decreased exploratory behavior in the light-dark box (LDB) [42, 50] (ii) ENK KO mice, exposed to stress induced by footshocks, present an anxiety-like behavior in LDB [52] (iii) DOPr KO mice spent less time in the illuminated portions of the LDB [53].
	Social interaction test	This test allows evaluating the propensity of an individual to socialize. The rodent is placed in an open-field arena alone in the first place and then with another individual. The time spent interacting with the intruder is measured.	(i) ENK KO mice present a reduced duration of social interaction [50].
	Forced swim test	This test is used to evaluate the antidepressant efficacy of new compounds. A rodent is placed in a pool containing approximately 15 cm^3^ of water, and its mobility is measured on a video-recording.	(i) Administration of a DOPr agonist increases mobility in the forced swim test [59].

Anhedonia	Sucrose preference test	This task is used as an indicator of anhedonia, characterized by a lack of interest for a reward. Two bottles, one containing a sucrose solution (between 1% and 5%) and another plain water, are presented to the animal. Its preference for the sweetened versus plain water reveals anhedonia state.	(i) After restraint stress, rats showing increased anhedonia (assessed with the sucrose preference test) present a reduced expression of ENK mRNA in the NAc [70].

**Table 2:** Evidence for ENK signaling involvement under different stress paradigms.

Paradigm	Principles and procedures	Evidence for involvement of ENK signaling
Single housing	Given the social behavior of rodents, chronic or acute single housing is used to mimic the stress due to social isolation. The animal is placed alone in its home cage.	(i) Prolonged single housing in early life decreases ENK immunoreactivity in AMG, SN, HPT, and PAG [63].

Restraint stress	The animal is placed in a tube in such a way that all movements are prevented. The psychological and physiological effects due to restraint stress result from the distress and aversive nature of the forced immobility.	(i) After acute restraint stress, ENK-degrading activity is reduced in AMG and increased in HPC [46] (ii) After chronic restraint stress, ENK *knockout* (KO) mice do not exhibit anxiety nor depression-like behavior [67] (iii) After chronic restraint stress, rats showing increased anhedonia present a reduced expression of ENK mRNA in the NAc [70].

Social defeat stress (or resident-intruder paradigm)	This task exploits the social conflict between two individuals to initiate psychological stress. This experiment can be related to the intimidation or victimization in humans. An intruder is placed in the home cage of a resident each day for a given period of time.	(i) After a chronic social defeat, *Oprm1* A112G mice show a strong resilience [71] (ii) After a chronic social defeat, resilient rats demonstrate a high recruitment of ENK afferents from PGi to LC [72] (iii) After a chronic social defeat in rats and mice, ENK mRNA levels decrease in BLA of vulnerable individuals [17, 81].

Chronic unpredictable stress	This test allows to mimic the unpredictable disruptions of daily life. An animal is subjected to different stressors each day for a given period of time. Stressors can include restraint stress, electric footschocks, wet bedding, group housing, mild shaking of the home cage, cold water swim, etc.	(i) ENK *knockdown* (KD) in BLA increases anxiety reproducing behavioral responses encountered in individuals vulnerable to chronic unpredictable stress [18].

## Abbreviations

DPDPE: [D-Pen 2,5]-enkephalin
TMT: 2,5-Dihydro-2,4,5-trimethylthiazoline
AMG: Amygdala
BLA: Basolateral nucleus of amygdala
BST: Bed nuclei of the stria terminalis
CNS: Central nervous system
CEA: Central part of amygdala
CRF: Corticotropin-releasing factor
EPM: Elevated plus maze
ENK: Enkephalin
FST: Forced swim test
HPC: Hippocampus
HPT: Hypothalamus
IC: Intercalated nuclei of amygdala
KD: *Knock down*
KI: *Knock in*
KO: *Knockout*
LDCVs: Large dense-core vesicles
CEAl: Lateral part of central amygdala
Leu-ENK: Leucine-enkephalin
LC: Locus ceruleus
MEA: Medial part of amygdala
mPFC: Medial prefrontal cortex
MEAP: Met-ENK-Arg^6^Phe^7^
Met-ENK: Methionine-enkephalin
NAc: Nucleus accumbens
OF: Open-field
LDB: Light-dark box
OPr: Opioid peptide receptor
PGi: Paragigantocellularis nucleus
PVN: Paraventricular nucleus of HPT
PAG: Periaqueducal grey
PET: Positron emission tomography
PTSD: Posttraumatic stress disorder
Pro-ENK: Proenkephalin
PKC-*δ*: Protein kinase C-*δ*
SI: Social interaction
SN: Substantia nigra
SR: Startle response
VTA: Ventral tegmental area
DOPr: *δ* opioid peptide receptor
KOPr: *κ* opioid peptide receptor
MOPr: *μ* opioid peptide receptor.
